# Superselective angiography of the wrist in patients with Kienböck’s disease

**DOI:** 10.1186/s12891-019-2492-5

**Published:** 2019-04-04

**Authors:** S. Kim, F. Eichenauer, A. Asmus, S. Mutze, A. Eisenschenk, P. Honigmann

**Affiliations:** 1 Abteilung für Hand-, Replantations- und Mikrochirurgie, Unfallkrankenhaus Berlin, Warener Str. 7, 12683 Berlin, Germany; 2 Institut für Radiologie und Neuroradiologie, Unfallkrankenhaus Berlin, Warener Str. 7, 12683 Berlin, Germany; 3 Klinik und Poliklinik für Unfallchirurgie, Universitätsmedizin Greifswald, Ferdinand-Sauerbruch-Straße, 17475 Greifswald, Germany

**Keywords:** Kienböck’s disease, Lunate, Superselective microangiography

## Abstract

**Background:**

Microvascular problems like increased intraosseous pressure or venous congestion may influence the development of Kienböck’s disease. We examined if wrist position modifies the blood flow in the nutrient vessels.

**Methods:**

Retrospective analysis of 17 patients with Kienböck’s disease who had a superselective microangiography of the radial, ulnar and interosseous artery in different wrist positions under general anaesthesia. We analysed the data with Fisher’s exact and Wilcoxon-test.

**Results:**

We found vessels that entered the bone, that ended at the bone edge, and that supplied a vascular plexus. The origins were the anterior interosseous artery in 10 of 17 cases, the radial artery in seven cases, and the ulnar artery in five cases. Movement of the wrist could reduce or stop the blood flow. Type of lunate configuration showed no significant influence on the blood supply in neutral position.

**Conclusion:**

The radial, ulnar, and anterior interosseous artery contribute to the vascular supply of the lunate bone in different combinations. Wrist movement can reduce blood flow to the lunate bone.

**Electronic supplementary material:**

The online version of this article (10.1186/s12891-019-2492-5) contains supplementary material, which is available to authorized users.

## Background

For the development of Kienböck’s disease, the avascular necrosis of the lunate, anatomical factors like ulnar variance, radial inclination, the number and location of nutrient vessels, and the venous drainage, or repetitive trauma may have an influence [1–3]. Using injection techniques and micro-CT it was possible to show the number, entry points, and anastomoses of nutrient vessels [3–6]. Wrist extension was shown to impair the venous flow of the lunate bone [7]. But these examinations were done ex vivo. Superselective angiography is a known method for the embolisation of tumours or vascular malformations or in case of bleeding [8–11]. We performed superselective angiographies in patients with Kienböck’s disease who were planned for surgical treatment to identify the blood vessels that contribute to the arterial perfusion of the lunate bone and present a qualitative assessment of the flow in different wrist positions.

## Methods

We retrospectively examined the patient files of patients presenting with Kienböck’s disease between 2009 and 2011 who were scheduled for operation. All patients had prior x-ray, CT, and MRI, confirming the diagnosis. We included 17 patients who received a superselective angiography of the lunate for preoperative planning. The angiography was used to determine the surgical approach that would the least compromise the vascular supply of the lunate.

Due to the radiation exposure during examination, angiography was performed only on the affected wrist for medical reasons, as there would be no consequence for the healthy side.

### Clinical and radiological assessment

Clinical and radiological values were recorded during outpatient clinic visits. Clinical information included age, gender, affected side, and hand dominance. From X-ray of both wrists, the ulnar variance, type of lunate, Stahl’s index, Carpal height according to Nattrass [12], and radial inclination were measured.

The stage of Kienböck’s disease according to Lichtman was determined by CT and MRI (Table 1).

**Table 1:** Patient information

	Sex	Affected side	Hand dominance	CT	MRI	Lunate type	Dose area product [cGy·cm^2^]	Fluoroscopy time [s]
1	M	Right	Right	IIIa	IIIa	I	–	–
2	M	Left	Right	IIIa	IIIa	II	1188.6	1217
3	M	Right	Right	IIIb	IIIb	I	905.8	564
4	M	Left	Right	IIIb	IIIa	I	1219.2	1148
5	F	Left	Right	IIIb	IIIb	I	413.7	841
6	M	Right	Right	IIIa	IIIb	I	–	–
7	M	Right	Right	II-IIIa	IIIa	I	338.7	445
8	F	Left	Right	IIIb	IIIb	I	223.6	307
9	M	Right	Right	IIIa	II	I	–	–
10	F	Right	Right	IIIa	IIIb	I	–	–
11	M	Right	Right	IIIb	IV	I	658.4	801
12	F	Right	Left	IIIa	IIIa	I	580.8	645
13	F	Left	Right	II	II	I	411.7	618
14	F	Left	Right	III	III	II	–	–
15	M	Right	Right	IIIa	IIIa	II	–	–
16	M	Left	Right	IIIa	IIIa	II	–	–
17	M	Right	Right	IIIb	IIIb	I	–	–

P: Patient number. Sex: M male, F female. Affected side: side of hand affected by Kienböck’s disease. Hand dominance: side of dominant hand. CT: Lichtman stage by CT. MRI: Lichtman stage by MRI. Type: Lunate type I: without a medial facet, II: with a medial facet. Dose area product in cGy·cm^2^, −: no data available. Fluoroscopy time in seconds

### Assessment of perfusion by angiography

The contrast agent flow in the radial, ulnar and interosseous artery was recorded under general anaesthesia in neutral position of the wrist. Assessment only of passive motion was possible. Entry point for the catheter was the femoral artery (angiography). Using a vertebral catheter, the axillary artery was entered (selective angiography) and contrast agent flow around the lunate examined after visualisation of the forearm arteries.

If no contrast agent was seen around the lunate, the examination was aborted. If it was seen, the radial, ulnar and interosseous arteries were subsequently entered (superselective angiography) with a micro catheter. If a contrast flow to the lunate bone was seen in neutral position, the flow in maximum passive flexion, extension, radial abduction, and ulnar abduction was classified as unchanged, reduced, or abolished. Branching of the vessel into capillaries around the capsule or synovial tissue that appeared as a cloud of contrast medium in close relationship to the lunate bone was regarded as blushing.

### Statistics

Due to the small sample size, non-parametric tests were used. Fisher’s exact test was used for categorical variables and Wilcoxon-test for quantitative variables. Significant differences were assumed for *p* < = 0.05.

## Results

We included six female and 11 male patients with a mean age of 35 years (median 31, range 19–64). Kienböck’s disease was located in ten right and seven left hands. The dominant hand was affected in 47% of cases (Table 1).

### Radiographic findings

Twelve patients had a negative ulna- variance with a maximum of four mm. Five patients had a neutral ulna variance. Comparisons between affected and control wrist are shown in Table 2.

**Table 2:** Radiographic parameters of the affected and contralateral side

	Stahl index		Carpal height [mm]		Ulnar variance [mm]		Radial inclination [degrees]	
	Kienböck	Control	Kienböck	Control	Kienböck	Control	Kienböck	Control
Median	0.35	0.47	1.42	1.47	−2.2	−1.8	25	23
Min	0.20	0.40	1.29	1.37	−5.6	−2.5	20	20
Max	0.50	0.61	1.64	1.76	0	+ 3.0	29	33

Stahl index, carpal height and ulnar variance are significantly different between affected and contralateral side (*n* = 17, *p* = 0.001, *p* = 0.001, *p* = 0.012, Wilcoxon-test), radial inclination showed no difference (*n* = 17, *p* = 0.164, Wilcoxon-test)

### Superselective angiography

Two lunate bones were supplied by the radial, ulnar, and interosseous artery. Seven lunate bones received blood from two of those arteries, and five by one nutritional artery. No contrast agent in the surrounding of the lunate bone was detectable in three patients (Patient 8, 11, and 16). The interosseous artery was in all cases identified as the anterior interosseous artery.

**Fig. 1:**
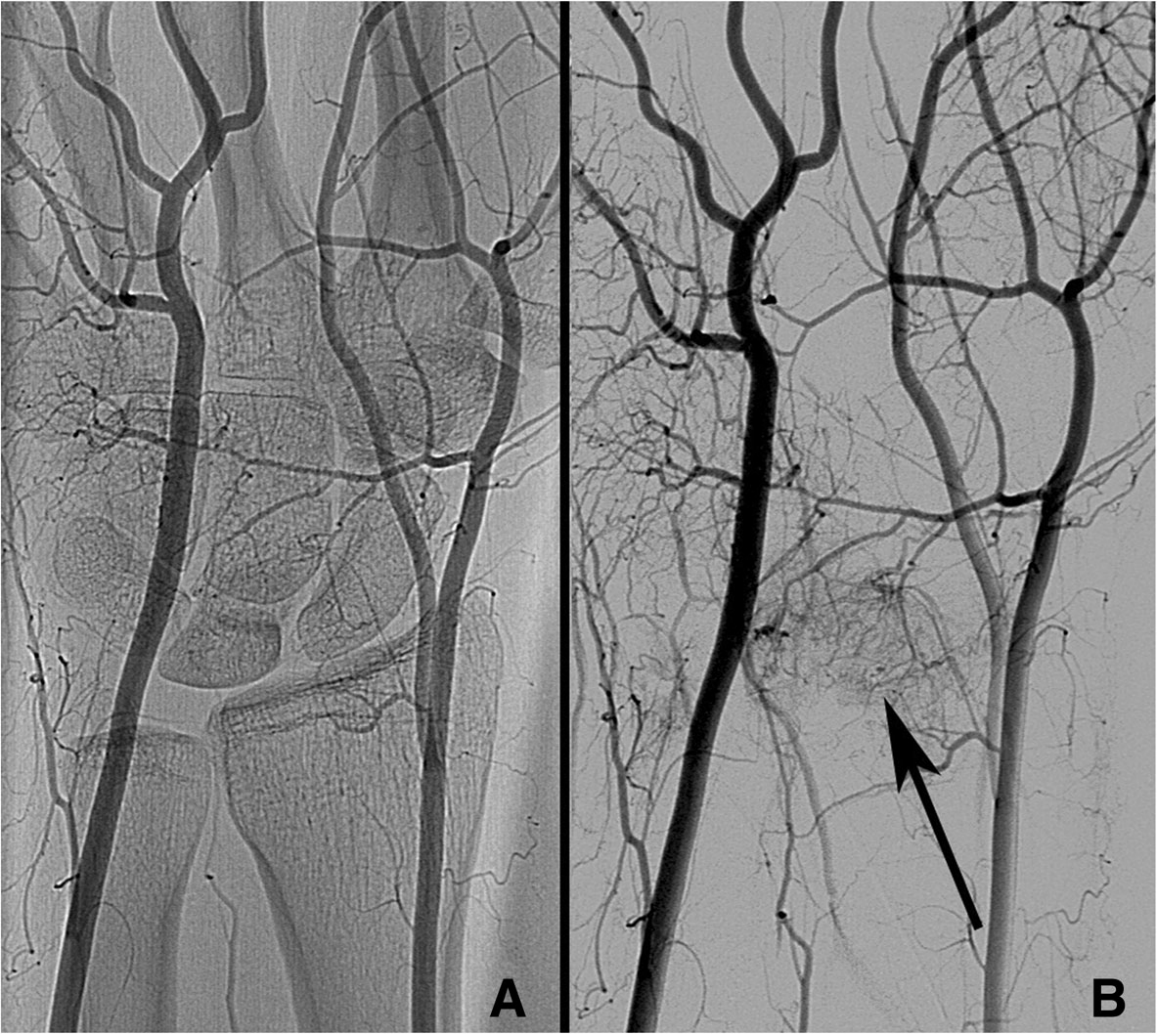
Angiography overview. **a** Angiography overview of a right hand with Kienböck’s disease Lichtman IIIa (patient 7). **b** the same hand with subtraction of bony structures, Arrow: capillary network (blushing) projecting onto the lunate and scapholunate ligament

**Fig. 2:**
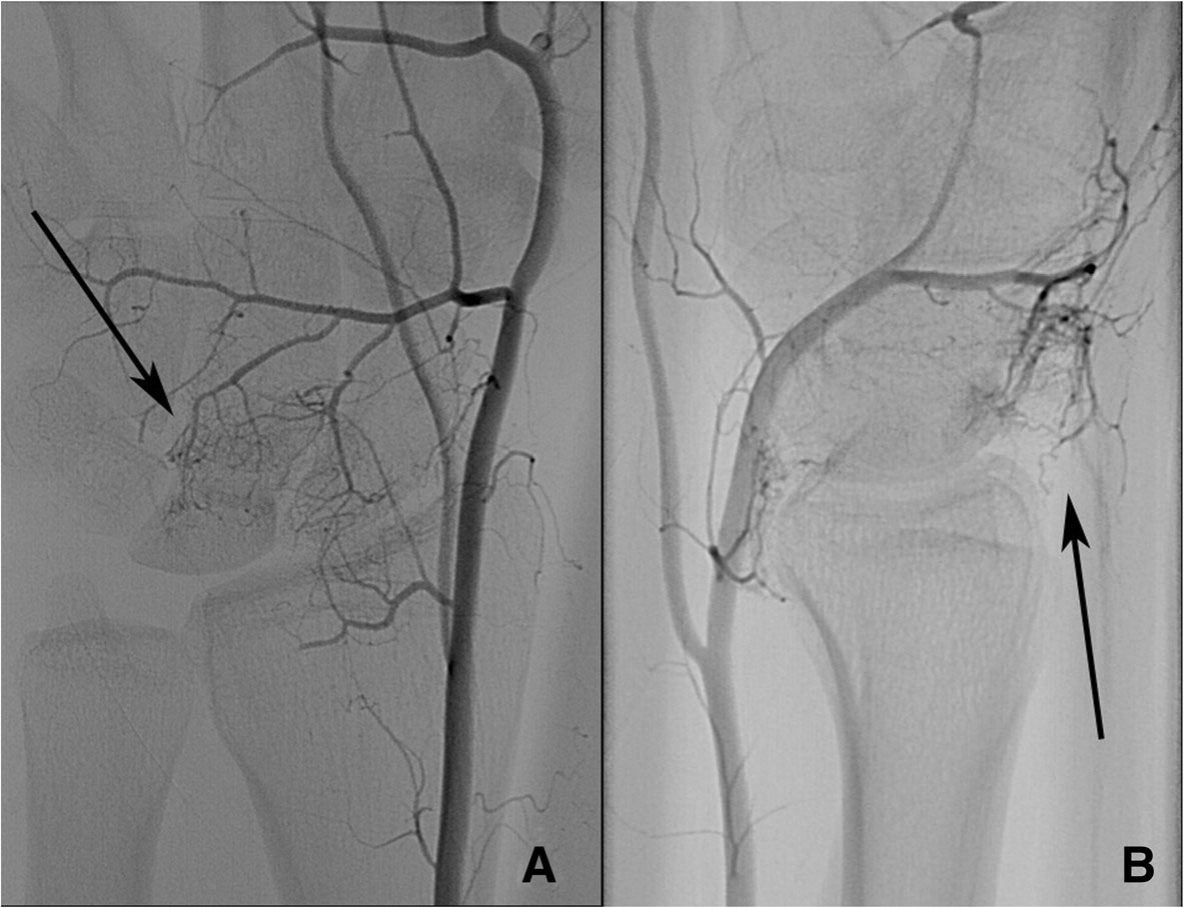
Angiography of the radial artery ap and lateral showing blush. Superselective angiography of the radial artery (patient 7). **a** ap view, **b** lateral view. Arrows: capillary network (blushing) with contact to the dorsal facet of the lunate

**Fig. 3:**
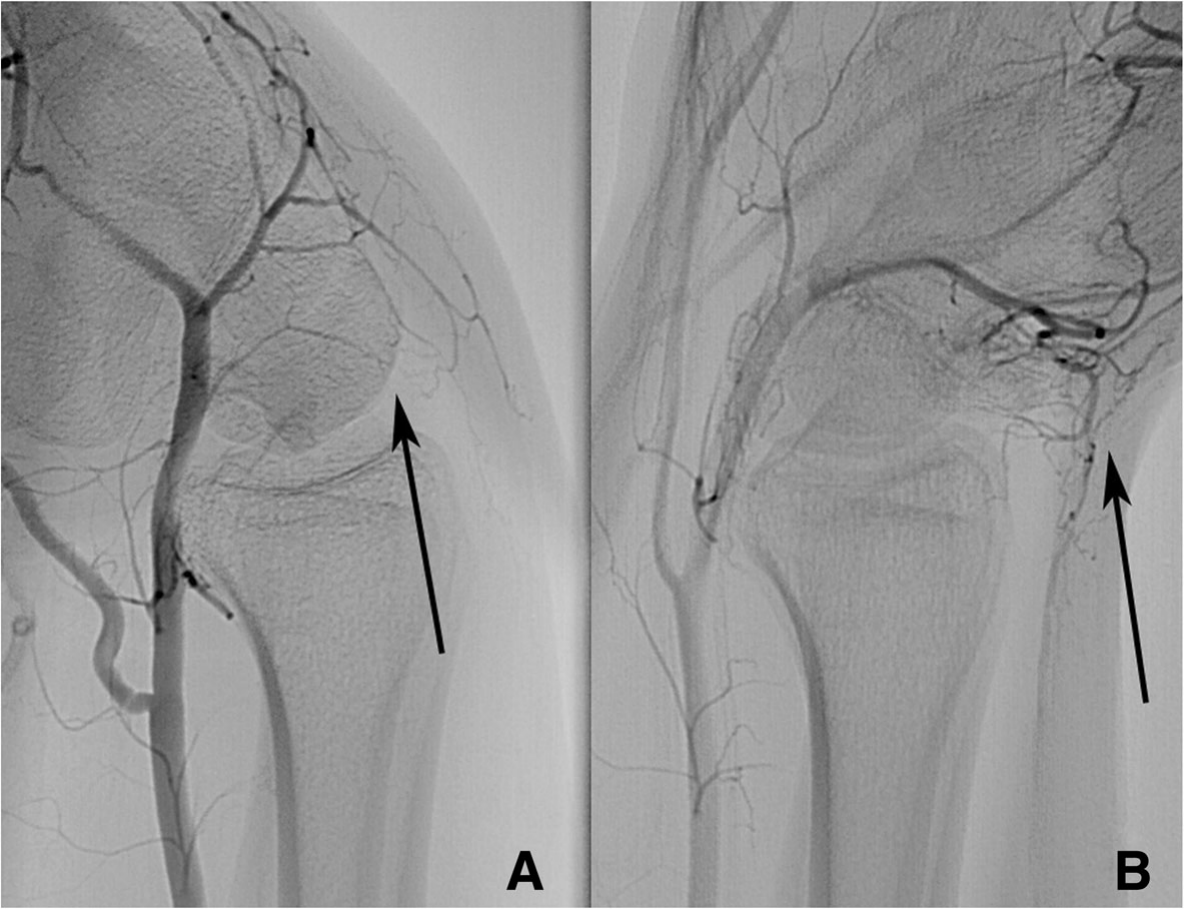
Superselective angiography of the radial artery shows stopped flow in flexion. Superselective angiography of the radial artery, lateral view (patient 7). **a** flexed wrist, **b** extended wrist. Arrows: The capillaries are not visible in flexion, but in extension

**Fig. 4:**
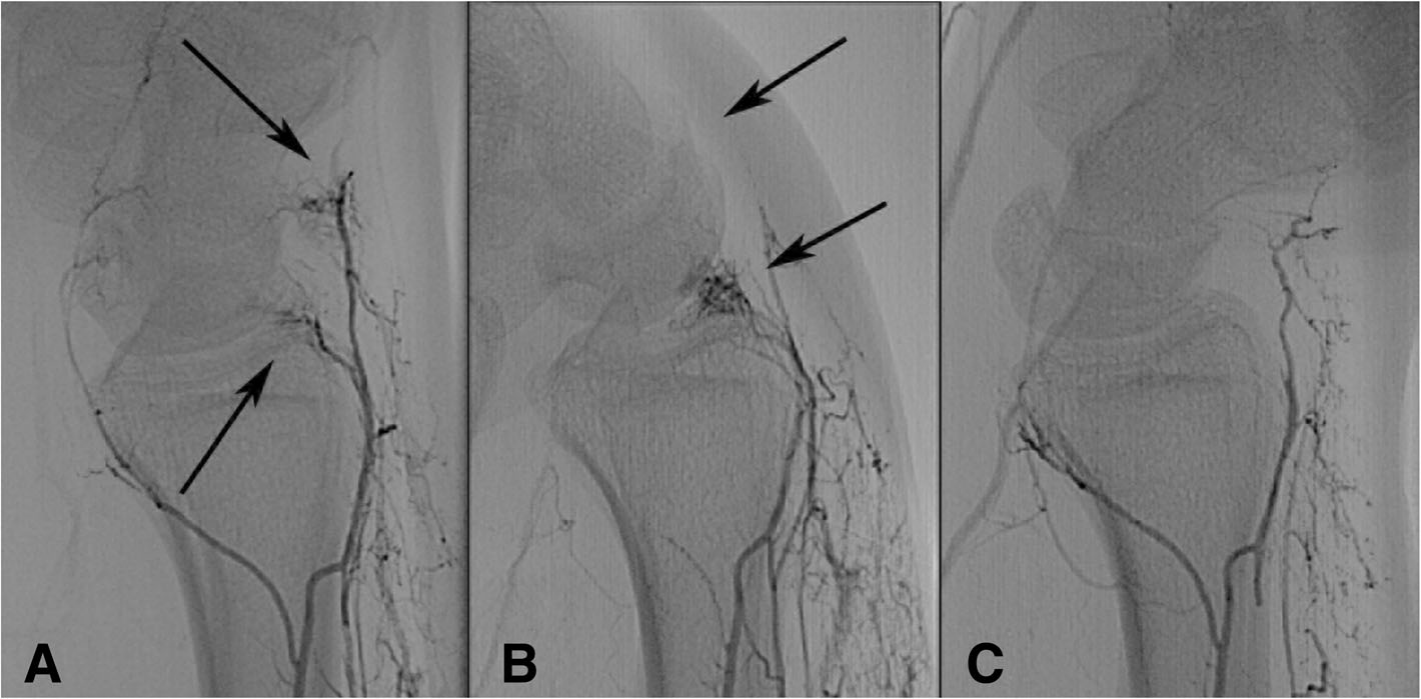
Superselective angiography of the interosseous artery show stopped flow in extension and flexion. Superselective angiography of the anterior interosseous artery, lateral view (patient 7). **a** normal position, **b** flexed wrist, **c** extended wrist. Arrows: Two blushing capillary areas are visible in the neutral position. In flexion the distal capillary area disappears, in extension both disappear


In neutral position of the wrist, the radial artery showed a vascular connection to the lunate in seven cases, the interosseous artery in eleven cases, and the ulnar artery in six cases of the remaining 14 patients (Table 3). Type of lunate configuration showed no significant influence on the blood supply in neutral position (Fisher’s exact, *p* > 0.05). In different wrist positions, blood flow can be disrupted (Figs. 1, 2, 3, 4 and Additional files 1, 2, 3, 4, 5, 6, 7 and 8, all from patient 7) but without discernible pattern (Fisher’s exact, *p* > 0.05). Due to the heterogeneity of the data and small sample size, no further statistical test was performed.

**Table 3:** Qualitative assessment of the vascular supply for each patient for each artery

P	Radial						P	Ulnar						P	Interosseous					
	Type	N	F	E	R	U		Type	N	F	E	R	U		Type	N	F	E	R	U
1	V	P	0	0	0	=	1	X						1	blush	P	–	–	–	–
	blush	D	0	=	0	↓		X							blush	D	–	–	–	–
2	X						2	blush	P	↓	↓	0	0	2	blush	P	=	=	=	=
	X							X							blush	D	↓	0	=	=
3	blush	D	0	0	0	=	3	X						3	blush	D	=	=	=	=
	blush	P	0	0	0	=		X							blush	P	=	=	=	=
4	V	D	↓	=	0	↓	4	X						4	V	P	↓	0	=	0
	V	P	↓	=	0	↓		X							V	D	=	↓	=	0
5	V	D	=	0	=	0	5	X						5	blush	P	0	0	=	↓
	blush	D	=	0	=	0		X							blush	D	0	↓	=	0
6	V	D	0	0	=	=	6	V	P	↓	↓	0	=	6	X					
	V	P	0	0	=	=		V	D	↓	↓	0	=		X					
7	blush	P	0	=	0	0	7	X						7	V	D	=	0	↓	0
	blush	D	0	=	0	0		X							X					
8	X						8	X						8	X					
9	X						9	V	P	0	0	0	0	9	X					
10	V	P	↓	–	↓	–	10	X						10	V	P	↓	↓	=	=
	V	D	↓	–	↓	–		X							X					
11	X						11	X						11	X					
12	X						12	blush	P	–	–	–	–	12	blush	P	=	↓	=	↓
	X							X							blush	D	=	↓	=	↓
13	X						13	X						13	blush	D	=	–	–	–
14	X						14	X						14	V	P	=	0	=	0
	X							X							V	D	↓	=	=	0
15	X						15	X						15	V	P	↓	↓	–	–
	X							X							V	D	↓	↓	–	–
16	X						16	X						16	X					
17	X						17	blush	P	–	–	=	↓	17	X					

P: Patient number, Type: type of vascular connection: X: no visible contrast agent around the lunate, V: vessel projecting into the bone, blush: contrast agent visible in capillary network surrounding the lunate. N: location of visible contrast agent in neutral position, possible locations: P: palmar and D: dorsal. F: in flexion, E: in extension, U: in ulnarduction, R: in radialduction. 0: no blood flow visible in contrast to neutral position, ↓: flow diminished compared to neutral position, =: unchanged to neutral position, −: no data available

Additional file 1:Angiography of the radial artery ap (Patient 7). (MP4 371 kb)



Additional file 2:Angiography of the radial artery lateral (Patient 7). (MP4 347 kb)



Additional file 3:Angiography of the radial artery lateral in extension (Patient 7). (MP4 310 kb)



Additional file 4:Angiography of the radial artery lateral in flexion (Patient 7). (MP4 261 kb)



Additional file 5:Angiography of the interosseous artery ap (Patient 7). (MP4 442 kb)



Additional file 6:Angiography of the interosseous artery lateral (Patient 7). (MP4 526 kb)



Additional file 7:Angiography of the interosseous artery lateral in extension (Patient 7). (MP4 398 kb)



Additional file 8:Angiography of the interosseous artery lateral in flexion (Patient 7). (MP4 245 kb)


## Discussion

Using superselective angiography, our findings show that there are different patterns of the blood supply of the lunate bone and that flow is influenced by the position of the wrist.

In previous studies, a vascular plexus located over the dorsal and palmar pole as well as direct branches from the ulnar, radial and anterior interosseous artery have been described that connect to the lunate [3, 13]. Dorsopalmar extraosseous anastomoses were observed [13, 14], that can be absent in 7.5% [15]. We found vessels that supplied the bone directly, vessels that seemed to end at the surface of the lunate and vessels that branched into a capillary network that resembled synovitis from either dorsal, palmar, or both sides that can be associated with bone necrosis [16].

The exact number of vessels entering the bone could not be counted in our study. But it was shown that micro-CT examination of the lunate showed significantly more nutrient foramina on the palmar side than on the dorsal side [6]. Numbers ranged from zero on the palmar side and one dorsally up to six on the palmar and nine on the dorsal aspect [6].

Our results showed a position-dependent blood flow of the nutritional vessels to the lunate in patients with Kienböck’s disease using superselective angiography. Except for the neutral position, every position could reduce the blood flow in every vessel. Table 4 shows that in almost all cases where supplying vessels could be identified, even if blood flow is stopped in one position from one artery, it is unchanged or only reduced in another if at least two arteries contribute to the blood supply. Intraosseous anastomoses between dorsal and palmar vessels (a common Y, and rarer I and X pattern) could be shown [4, 17], therefore disruption of blood supply might not occur in healthy subjects. As fracture of the bone or increased intraosseous pressure and venous congestion might disrupt the anastomoses [18–20], holding certain wrist positions for a prolonged time could contribute to the progress of the disease. Rarefication of the intraosseous trabecular structure could be shown by in vivo high-resolution peripheral quantitative micro-CT of an affected wrist with a recovery of trabecular parameters after radial shortening osteotomy [21]. If the structural changes are a result or cause of the disruption of intraosseous blood flow would need a combined diagnostic approach.

**Table 4:** Summarised effect of wrist position on blood flow

P	Wrist position			
	Flexion	Extension	Radialduction	Ulnarduction
1	0	= r	0	= u
**2**	= i	= i	= i	= i
**3**	= i	= i	= i	= i
**4**	= r/i	= r	= i	↓ r
**5**	= r	↓ r/i	= r/i	↓ i
**6**	↓ u	↓ u	= r	= r
7	= i	= r	↓ i	0
9	0	0	0	0
**10**	↓ r/i	↓ i	= i	= i
**12**	= i	↓ i	= i	↓ i
14	= i	= i	= i	0

Only patients with available information for each wrist position are listed. For each position, the artery with the least impairment is shown. r: radial artery, u: ulnar artery, i: interosseous artery. 0: no blood flow visible, ↓: flow diminished compared to neutral position, =: unchanged to neutral position, −: no data available. P: patient number, F: in flexion, E: in extension, U: in ulnarduction, R: in radialduction. In seven of eleven patients marked in bold, the blood flow is maintained in any wrist position

We decided to regard the synovial blushing as contributing to the blood supply, because of the high number of foramina that would be entered by vessels from the dorsal and palmar plexus and the intraosseous anastomoses. A summarised version of Table 3 is shown in Table 4, showing the least compromising effect of wrist positioning, and assuming that microanastomoses provide sufficient blood distribution.

In three patients, no flow to the lunate was visible, suggesting none or minimal capillary blood supply to necrotic tissue. As the lunate stage was IIIa, IIIb, and IIIb/IV, it can only be assumed that the stages IIIa and b were diagnosed before further, maybe rapid progression or the vessels were too fine to be seen.

It is inconclusive if Kienböck’s disease is caused by hand-arm vibration that occurs during manual work with percussive tools [22]. But tool manufacturers might consider these results and provide a design that avoids extensive wrist flexion or extension. Splinting in neutral position might be better for blood flow than dorsal extension in cases with Kienböck’s disease.

In most cases, the interosseous artery was contributing to the blood flow of the lunate bone. Performing a selective wrist denervation of the interosseous nerve might influence the operative outcome due to reduced blood flow due to scarring or thermal damage when using electrosurgery. Recent articles recommend a radical denervation for the treatment of chronic wrist pain [23–26]. Considering that this procedure is rather common, we found no report of an increase of cases with Kienböck’s disease [27, 28].

Our radiographs showed a larger radial inclination of the control side compared to the affected side that is not significant. In previous studies, the degree of radial inclination was significantly larger in the unaffected wrist of patients with Kienböck’s disease [1, 13]. Our findings are in the range of the previously reported values. As the difference is rather small, the number of cases could be too small to show a significant difference.

Stahl’s index and carpal height was significantly lower on the affected side, corresponding to a carpal collapse. But also the unaffected wrist showed smaller values compared to the reference in the literature for Stahl’s index (0.53 ± 0.03) and carpal height (1.52–1.62) [12]. The incidence of lunate type I was higher in our sample with 76% compared to previously reported numbers [3].

Weaknesses of our study include the low number of examined patients and the lack of a healthy control group. In cases without contrasted vessels to the lunate, the vessels might have been too small to visualise or the moment of staining missed. In addition, reduced blood flow by stretching of capillaries during wrist movement might by a physiological phenomenon. Examination of healthy hands might be more conclusive but are not ethically acceptable due to high radiation exposure. The national Federal Office for Radiation Protection does not give a reference for arm angiography but our examinations were below the value for arteriographies of pelvis and leg that should not exceed 4800 cGy x cm^2^ [29]. Finally, without 3 D reconstruction, we can only assume that the vessels are entering the bone.

## Conclusion

The radial, ulnar, and anterior interosseous artery contribute to the vascular supply of the lunate bone in different combinations. Wrist movement can reduce blood flow to the lunate bone. Prolonged flexion or extension of the wrist due to splinting, pain or occupational reason might contribute to the development of Kienböck’s disease.
